# Examining the dimensionality of morphological knowledge and morphological awareness and their effects on second language vocabulary knowledge

**DOI:** 10.3389/fpsyg.2023.1207854

**Published:** 2023-08-11

**Authors:** Tuoxiong Wang, Haomin Zhang

**Affiliations:** ^1^School of Foreign Languages, Ningxia Normal University, Guyuan, Ningxia, China; ^2^The Psycholinguistics Lab, School of Foreign Languages, East China Normal University, Shanghai, China

**Keywords:** morphological knowledge, morphological awareness, vocabulary knowledge, dimensionality, mediation analysis

## Abstract

Morphological knowledge and morphological awareness are multidimensional and both have been confirmed to make important contributions to vocabulary knowledge. However, the extant literature has not made a clear demarcation between morphological knowledge and morphological awareness. The current study examined the underlying components of morphological knowledge and morphological awareness as well as their effects on vocabulary knowledge. The performance of 226 tenth- and eleventh-graders on five tasks was investigated using confirmatory factor analysis and structural equation modeling. Results demonstrated that morphological knowledge and morphological awareness were two distinct constructs. In regard to the direct and indirect effects between morphological knowledge and vocabulary, it was indicated that morphological knowledge made a significant indirect effect on vocabulary knowledge through morphological awareness. However, the direct effect of morphological knowledge on vocabulary knowledge was not significant. Findings from the current study have important implications to adolescent EFL students’ vocabulary instruction and research.

## Introduction

Morphemes are the smallest meaningful units of language which determine how well novel and complex words are learned (Jackson and Zé Amvela, 2021). Morphological knowledge has been confirmed to contribute to different aspects of literacy development such as vocabulary growth (e.g., Anglin, 1993; Carlisle and Fleming, 2003; Nagy et al., 2006). Studies have evidenced that a surge growth of vocabulary in fourth grade is attributable to affixed words, that is, words are formed through morphemes of prefixes, suffixes or both (Nagy and Anderson, 1984; White et al., 1989). The ability to consciously use morphological knowledge to derive unfamiliar word meanings becomes especially important for adolescent students because they have been placed heavier burdens on academic language which is characterized by considerable morphologically complex words. Both morphological knowledge and morphological awareness have been considered as robust predictors of vocabulary growth in the current literature (e.g., Nagy et al., 2006; Larsen and Nippold, 2007). However, prior studies have not made a clear distinction between morphological knowledge and morphological awareness. Whether morphological awareness and morphological knowledge are the same or separate constructs remains a question. Few studies have tapped into the constructs of morphological knowledge and morphological awareness using confirmatory factor analysis (CFA). In addition, morphology has been highlighted in middle and high school instruction for the expansion of vocabulary, which is explicitly specified in General Senior High School Curriculum Standards (2017 edition) developed by the Ministry of Education of the People’s Republic of China (Ministry of Education, 2001). Yet the existing literature has scarcely focused on the relationship between morphological knowledge and vocabulary knowledge in adolescent EFL learners. Taken together, the present study aimed to conduct research from two aspects in light of the research gaps mentioned above. It firstly intended to verify the constructs of morphological knowledge and morphological awareness by using CFA because the boundaries between morphological knowledge and morphological awareness mainly depended on how these concepts were conceptualized and assessed (Goodwin et al., 2021). Drawing upon structural equation modeling (SEM), the current study also aimed to examine the relation between morpheme form, morpheme meaning and morpheme use loaded on the latent variable of morphological knowledge (or awareness) and vocabulary knowledge in the form of observed variables of vocabulary size and vocabulary depth to have accurate assessment with measurement errors, which can supplement the methodological limitations in previous studies.

### Morphological knowledge and morphological awareness

In view of the definition of morphological knowledge, the existing literature has not presented a clear picture. Morphological knowledge was depicted as less conscious or implicit processing of morphological information, and it was interchangeable with morphological processing (e.g., Deacon et al., 2008). However, Nagy et al. (2014) stated that morphological knowledge was used as an umbrella term covering morphological awareness and morphological processing. Their definition was aligned with Goodwin et al., 2021, indicating that morphological knowledge was multidimensional encompassing morphological awareness and information conveyed by specific morphemes such as prefixes, suffixes, and roots. Compared to the definition of morphological knowledge, Carlisle’s (1995) definition of morphological awareness has been well-acknowledged within research and practice. It refers to “awareness of the morphemic structure of words and the ability to reflect on and manipulate that structure” (p. 194). In other words, morphological awareness pertains to the abilities to break down words into smaller meaningful units such as prefixes, suffixes and roots, and to derive meanings of new words from smaller morphemic parts. Based on the conceptualization above, we could conclude that morphological knowledge mainly concerned implicit acquisition of the principles of word formation as well as the knowledge of specific morphemes whereas morphological awareness was defined as the conscious reflection on and manipulation of morphemic structure. Carlisle (2004) indicated that without the knowledge of word parts such as specific knowledge of prefixes and suffixes, to consciously process morphologically complex words was impossible. However, in the extant literature, morphological knowledge and morphological awareness have been measured with the same or similar tasks which might disguise the demarcation of these two constructs (e.g., Carlisle, 2000; Muse, 2005; Foorman et al., 2012; Kirby et al., 2012; Deacon et al., 2014; Zhang, 2015). For example, Carlisle (2000) used morpheme derivation task to assess morphological awareness while Foorman et al. (2012) and Muse (2005) utilized the same task to capture morphological knowledge. To our knowledge, morphological knowledge in current literature thus far was not assessed with the knowledge of specific morphemes, which was overlooked in previous studies. As Goodwin et al. (2021) stated that the constructs of morphological knowledge and morphological awareness were determined by how they were measured. Goodwin et al. (2017) examined the multidimensionality of morphological knowledge with seven morphological tasks among 371 seventh- and eighth-graders. Based on CFA, the findings showed that morphological knowledge was best fit by a two-factor model represented by a general factor of morphological knowledge, which was consistent with findings of Muse (2005) and Tighe and Schatschneider (2015). Similarly, Muse (2005) attempted to investigate the underlying nature of morphological knowledge, that is, to test the hypothesis that morphological knowledge can be categorized into two dimensions of morphological awareness and morphological knowledge. The results demonstrated that the two subcategories of morphological knowledge were not theoretically separate, and they were best represented with a unidimensional construct of morphological knowledge. As such, Tighe and Schatschneider (2015) conducted a study on the construct and potential multidimensionality of morphological awareness in Adult Basic Education (ABE) students with three sets of different measures, namely inflected versus derived, real words versus pseudowords, and contextual cues versus no contextual cues. The results indicated that dimensions of morphological awareness varied from the measured tasks. For example, the facets of inflectional and derivational tasks were not confirmed as separate latent dimensions of morphological awareness while real words and pseudowords tasks represented two separate facets. Such results were also verified in Levesque et al. (2017) study, showing that multidimensional morphological awareness depended on the type of measurement tasks. According to the aforementioned studies, morphological awareness was considered as one subcategory of morphological knowledge. Additionally, the tasks used to measure morphological awareness or morphological knowledge focused on the ability to analyze word structures from morphemic units. There was a dearth of studies on assessing students’ abilities to identify and to recognize the form, meaning and grammatical functions of specific prefixes and suffixes within words, which are the core of morphological knowledge or morphological awareness. To this end, the present study aimed to test uni-construct or bi-construct models represented by the latent variable of morphological knowledge or morphological awareness.

### Relations between morphological knowledge/morphological awareness and vocabulary knowledge

On the basis of preceding studies, there were no clear distinctions between morphological knowledge and morphological awareness. To reduce wordiness, the current discussion used morphological awareness to refer to the two concepts.

Morphological awareness is characterized as a meta-linguistic ability which encompasses procedural knowledge about words and the rules that govern word formation whereas vocabulary knowledge is portrayed as a linguistic ability which involves declarative knowledge of words (Kuo and Anderson, 2006). The combination of linguistic and meta-linguistic abilities is critical to literacy outcomes. Morphological awareness helps children grasp and memorize the meaning of vocabulary through discriminating morphemes and analyzing morphological structure of vocabulary (McBride, 2016). It has been documented that morphological awareness makes considerable contributions to the development of vocabulary knowledge. Sepcifically, Nation (2013) states that 8,000–9,000 word families are the optimal lexical threshold in reading comprehension. To be more precise, learners need to know 40,000 word types in order to achieve this optimal vocabulary threshold. Without reliance on morphological awareness to access the meaning of novel derived words in reading, it is demanding for second or foreign language learners to grasp 8,000–9,000 word families. Additionally, every year average upper elementary students encounter about 10,000 new words which they have not previously encountered in print; moreover, more than half of all English words are morphologically complex (Nagy and Anderson, 1984). The importance of the knowledge of morphemes as well as the the ability to reflect on and manipulate morphemic structure have also been confirmed in Goodwin and Perkins (2015), indicating that a large proportion of words occurring in textbooks are morphologically complex words which are formed through adding prefixes or suffixes to lexical morphemes (root words) to create new words.

Regarding the relation between morphological awareness and vocabulary, studies on monolingual English speakers have provided evidence that morphological awareness is strongly related to vocabulary knowledge (Schmitt and Meara, 1997; Mochizuki and Aizawa, 2000; Sasao, 2013). Anglin (1993) indicated that new derived words were learned three times faster than new root words in first and fifth graders. The growth in derived word knowledge corresponded to the increasing use of derived words in written texts starting in later elementary years (White et al., 1989). It was found that 60% of new words middle school students met in textbooks in school were transparent and derived words whose meanings could be retrieved by analysis of morphemic structure (Nagy and Anderson, 1984); therefore, structural awareness of morphologically-complex words plays a critical role in vocabulary learning. The extant literature has empirically established the robust relationship between morphological awareness and vocabulary knowledge (Berko, 1958; Anderson and Freebody, 1983; Anglin, 1993; Carlisle, 2000; Ku and Anderson, 2003; Nagy et al., 2006; Zhang and Koda, 2012). As a case in point, it was corroborated that fourth graders’ morphological awareness strongly correlated with their vocabulary knowledge when orthographic awareness and phonological awareness were taken into account (Nagy et al., 2003). Nagy et al. (2006) also reported that morphological awareness and vocabulary knowledge were highly correlated for fourth- and fifth-grade students. Studies have shown that the association between morphological awareness and vocabulary knowledge increases with grade levels, and the relationship tends to be strengthened from first and second grades (Carlisle, 1995), to middle elementary grades (Fowler and Liberman, 1995) and high schools (Mahony, 1994). For example, Wysocki and Jenkins (1987) explored students’ ability of using morphological knowledge to derive meanings of unfamiliar words in fourth, sixth, and eighth graders. The results revealed that the older graders performed much better than younger graders in word-meaning retrieval. In alignment with Wysocki and Jenkins’ study, Tyler and Nagy (1989) discovered that relational and syntactic knowledge of morphology increased across grade. Similarly, Windsor (1994) found that cognitively mature students were advantageous at comprehending and producing derivational suffixes. The aforementioned studies centered on the relationship between morphological awareness and vocabulary knowledge among monolingual English speakers. By the same token, a handful of studies have investigated the contribution of morphological awareness to vocabulary acquisition among English as a Second Language (ESL) or as a Foreign Language (EFL) learners. Schmitt and Meara (1997), examined the correlation between vocabulary knowledge, morphological knowledge, and lexical associative knowledge in 95 Japanese middle and high school students and demonstrated that morphological knowledge was moderately correlated with lexical associative knowledge and lexical breadth. Kieffer and Lesaux (2012) further investigated the growth of morphological awareness and its effects on vocabulary knowledge in Spanish-speaking English language learners. The findings substantiated that learners’ morphological awareness developed with grade levels, and learners’ vocabulary knowledge grew with morphological awareness. However, the mechanism between the morphological awareness and vocabulary knowledge is complicated. More specifically, a mediator may associate morphological awareness with vocabulary knowledge. Zhang and Koda (2012), for instance, probed into the direct and indirect effects of morphological awareness on L2 vocabulary knowledge *via* the mediation of lexical inferencing. The findings verified that morphological awareness made both direct and indirect contributions to vocabulary knowledge through the mediation of lexical inferencing. Therefore, the mediated and unmediated relationship needs further exploration.

Taken together, morphological knowledge in present study was conceptualized as the implicit processing of principles of word formation as well as the knowledge of specific morphemes such as how they convey semantic and syntactic meaning whereas morphological awareness was conceptualized as the conscious awareness of word structures and the ability to reflect on and manipulate that structure. However, the tasks utilized to test these two constructs primarily drew on measures such as word analogy task, derivation and decomposition tasks which were well-acknowledged in the assessment of morphological awareness or morphological knowledge. In addition to studies exploring morpheme acquisition (Carlisle, 2000; Nagy et al., 2014), few studies have examined morphological knowledge/or morphological awareness with specific knowledge of morphemes (prefixes and suffixes) and its effect on vocabulary. The current study was designed to verify whether morphological knowledge and morphological awareness were two separate constructs when the knowledge of specific morphemes of prefixes and suffixes were included in the measurement tasks, which was not touched on in prior literature. Meanwhile, regarding the methodological limitations in previous studies, measurement errors underestimated the magnitude of mediated effect and overestimated the strength of the direct effect (Hoyle and Kenny, 1999). In order to improve the accuracy of mediated effect measurement, the latent variable models which are specified as the true measure of construct (MacKinnon, 2008) are used in present study aiming to delve into the underlying mechanism between latent variables in structural modeling analysis.

Therefore, the present study intended to fill the research gap by addressing two following research questions.

1. Are morphological knowledge and morphological awareness separate constructs or the same construct among Chinese adolescent EFL students?
2. Are there direct and indirect routes associating morphological knowledge, morphological awareness and vocabulary knowledge?

## Materials and methods

### Participants

Participants were 226 (117 tenth graders; 109 eleventh graders; 106 males, 120 females) who were recruited from one senior high school in the northeastern China. The school reported grades of A on Ningxia standardized tests (Zhongkao) and the participants’ English scores ranged from 100 to 120 (120 being the maximum score). Participants had two-session English classes each day. The English instruction has been conducted on the basis of General Senior High School English Curriculum Standards (2007 edition) stipulated by the Chinese Ministry of Education (MOE), which recommends vocabulary being taught through the knowledge of word formation (i.e., root and affix knowledge) to expand vocabulary.

### Measurements

Vocabulary knowledge is categorized into receptive and productive knowledge with the former referring to the language skills of listening and reading, and the latter concerning language skills of speaking and writing (Nation, 2022). The existing literature has empirically verified the unidimensionality of vocabulary knowledge in second language learning (Gonzalez-Fernandez, 2022; Ha and Nguyen, 2023), which lends the support to the present study to explore the receptive vocabulary knowledge including the breadth and depth of word knowledge (Read, 1993). Vocabulary Size Test (VST) and the Word Associate Test (WAT) were used to test the two aspects, respectively.

### Vocabulary size

Participants’ vocabulary size was measured with the Vocabulary Size Test (VST) developed by Nation and Beglar (2007). There were 140 items chosen from 1st 1,000 to 14th 1,000 word-family levels. The target word was presented in English and the four choices were in Chinese. According to participants’ English language level, the 1st 1,000 to 6th 1,000 word levels were used in present study. Participants were asked to choose the correct meaning of each target word from four definitions. For instance, the sentence *They saw it.* was shown to the participants, and they were required to select the most appropriate word meaning from four options: (a). 切 *cut* (b). 等待 wait (c).看 *look* (d). 开始begin. The reliability (Cronbach α) was 0.76.

### Vocabulary depth

Participants’ depth of vocabulary knowledge was assessed using the Word Associate Test (WAT) (Read, 1993). There were 40 items followed by two boxes of four words. To be specific, WAT was designed to test two aspects of the knowledge of vocabulary depth: meaning and collocation. A target word was followed by eight other words with different word classes. The four adjectives were assigned in the left box and the other four nouns were in right box. One to three words on the left were synonyms of the target word, and one to three words on the right were collocates of the target word. Participants were required to choose four correct words from two boxes, and they were informed that the four correct answers were not evenly spread in two boxes. As illustrated in the example below, participants need to choose *A. clever C. happy D. shining* from the left box, and *A. color* from the right box. The reliability (Cronbachα) was 0.83.

Bright.

**Table:** 

clever B famous C. happy D. shining	color B. hand C. poem D. taste

### Morphological knowledge

Morphological knowledge in current study refers to the rules of word formation and the implicit knowledge of specific morphemes. It was measured with the Word Part Levels Test (WPLT) adapted from Sasao and Webb (2017). The WPLT includes 118 affixes (42 prefixes; 76 suffixes) which were singled out from 10,000 word families in a word list of British National Corpus (BNC) developed by Nation (2012). The test was validated and categorized into three levels: beginner, intermediate and advanced levels with 40, 39, and 39 affixes, respectively. The affixes in each level were measured from three sections: morpheme form, morpheme meaning and morpheme use. According to participants’ English language proficiency, we chose the intermediate level test. The reliability (Cronbach α) of three sections were 0.89, 0.73, and 0.76, respectively.

### Morpheme form

The morpheme form section involves the recognition of written affixes, including 37 items. Each item was shown with four alternative morphemes appearing in the same position in the word with the same number of letters. Participants were asked to select one of the four alternative morphemes, such as *-ing*, −*nge*, −*eld,* and -*kle*, that could change the word’s meaning or the part of speech of the word base.

### Morpheme meaning

The morpheme meaning section aims to test knowledge of receptive affix meanings. There were 21 affixes and the each item was presented with two example words that contained the affix. Participants were required to choose the meaning of the target affix from four choices as shown in example *-ed* (walked; played) (1) *past* (2) *not* (3) *many* (4) *person.*

### Morpheme use

Morpheme use aims to measure knowledge of the part of speech carried by affixes. There were also 21 items and the each affix was presented with two example words from noun, verb, adjective, and adverb. For example, participants had to choose correct part of speech of affix *-ed* attached to two words *walked* and *played* from four choices of (1) Noun (2) Verb (3) Adjective (4) Adverb.

### Morphological awareness

Participants’ morphological awareness was measured with two tasks of morpheme recognition and morpheme discrimination (Ku and Anderson, 2003), aiming to test participants’ conscious awareness of word morphemic structure and their ability to reflect on and manipulate that structure.

### Morpheme recognition

A morpheme recognition task was used to test participants’ ability to recognize the morphological relationships between pairs of words. Participants were shown 20 pairs of words followed by *yes* and *no,* and asked to indicate whether the second word “comes from” the first word. For example, participants needed to decide whether the word *teacher* comes from the word *teach*. The reliability (Cronbach α) was 0.81.

### Morpheme discrimination

The morpheme discrimination test was constructed to examine participants’ ability to distinguish English compound structures. The test consists of 20 groups of words. Each group has three words that share a part. Participants were asked to circle the odd word from each group. For example, among the words *classroom, bedroom* and *mushroom*, the last word is odd because the meaning of the word *room* in *mushroom* is different from the meaning in the other two words *classroom* and *bedroom*s. That is, the *room* in the first two words means a physical building constructed with walls, floors and ceiling whereas the *room* in *mushroom* is a monomorphemic word which does not denote the morphemic meaning of *room*. The reliability (Cronbach α) was 0.78.

### Procedures

Five paper-and-pencil tests were administered to the participants in a whole class session by their teachers. To avoid potential confound of participants’ understanding of task instructions, we provided detailed Chinese directions prior to the administration of each task. The morphological knowledge measurements were first administered in the first week, followed by morphological awareness tests, and finally the vocabulary knowledge tests. The duration of data collection lasted for 1 month. Sufficient time was allowed for all participants to complete each measure.

### Data analysis

To address two research questions, we first used SPSS 23.0 to calculate the indicators of descriptive statistics such as means and standard deviation as well as the correlations between all observed variables. We then conducted confirmatory factor analysis (CFA) and structural equation modeling (SEM) using Amos Version 26 to answer two research questions. The CFA was used to test the hypothesized measurement model and the SEM was used to test hypothesized structural models. To be specific, CFA was adopted to answer the first research question concerning whether morphological knowledge and morphological awareness were distinct or same constructs, and SEM was to answer the second research question involving the correlations between latent variables. CFA is theory-driven tapping into the dimensionality of constructs over exploratory factor analysis (Bollen, 1989), which involves the specification and estimation of one or more hypothesized models of latent factors (Bagozzi, 1980; Joreskog and Sorbom, 1989). On the basis of the previous literature on the dimensionality of morphological awareness/morphological knowledge, two hypothesized models were constructed: one was single-factor model and another was two-factor model. The goodness-of-fit indices recommended in the literature were evaluated in the current study to compare and evaluate measurement and structural models. The cut-off values for different indices were proposed: Comparative Fit Index (CFI) and Tucker-Lewis Index (TLI) larger than 0.95, as well as Root Mean Square Error of Approximation (RMSEA) smaller than 0.08 (Hu and Bentler, 1999). If morphological knowledge and morphological awareness were confirmed as two distinct constructs, the indirect effects of morphological knowledge/or morphological awareness on vocabulary knowledge were tested. That is, we were interested in whether morphological awareness served as mediators for the effects on vocabulary knowledge. In order to avoid errors that were commonly overlooked in traditional multivariate procedures, we also used bias-corrected (BC) bootstrapped 95% confidence intervals to evaluate the statistical significance of the direct and indirect effects of interest, which complemented the limitations produced by other significance tests (Preacher and Hayes, 2008).

## Results

Table 1 shows the results of descriptive analysis including the mean, range, standard deviation and normality testing. Compared with the values of standard deviations for other variables, the standard deviations for vocabulary size and vocabulary depth showed a relative wide dispersion. The skewness values of each variable ranged from 0.04 to −0.94, and the kurtosis values ranged from −0.02 to −1.18, which conformed to the principle that the absolute value of skewness is less than 2 and the absolute value of kurtosis is less than 7 (Kline, 2005). The kurtosis and skewness indices signified that the data set was normally distributed. Table 2 presents the Pearson correlation coefficients of all variables. All the measures were significantly and positively correlated (*p*s < 0.01). According to Cohen’s (1988) rules of thumb, correlations above 0.3 indicates medium and correlations above 0.5 are high. In light of this, the correlations of vocabulary size and vocabulary depth with all other variables were medium or high, with exception of morpheme form.

**Table 1:** Descriptive statistics for all measures.

Measure	*N*	Min	Max	Mean(SD)	Skewness	Kurtosis	Item total
Morpheme form	226	6	37	23.4(7.48)	0.04	−1.18	37
Morpheme meaning	226	1	21	14.42(4.15)	−0.91	0.54	21
Morpheme use	226	2	21	14.58(4.16)	−0.94	0.53	21
Recognition	226	9	20	16.27(2.01)	−0.45	0.27	20
Discrimination	226	6	20	15.28(2.84)	−0.54	−0.02	20
Vocabulary size	226	19	60	39.24(9.43)	0.48	−0.52	60
Vocabulary depth	226	11	76	53.35(10.00)	−0.76	1.96	80

**Table 2:** Correlations among all measures (*N* = 226).

Measure	1	2	3	4	5	6	7
Morpheme form	–						
Morpheme meaning	0.488**	–					
Morpheme use	0.508**	0.640**	–				
Recognition	0.222**	0.418**	0.340**	–			
Discrimination	0.394**	0.422**	0.441**	0.453**	–		
Vocabulary size	0.272**	0.491**	0.455**	0.368**	0.343**	–	
Vocabulary depth	0.333**	0.492**	0.528**	0.433**	0.436**	0.455**	–
All are significant at **p < 0.01.							

### Measurement model

**Figure 1:**
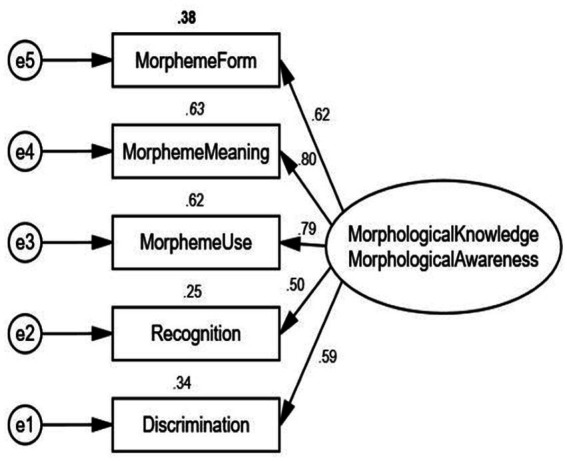
Single-factor confirmatory analysis of morphological knowledge/or morphological awareness.

**Figure 2:**
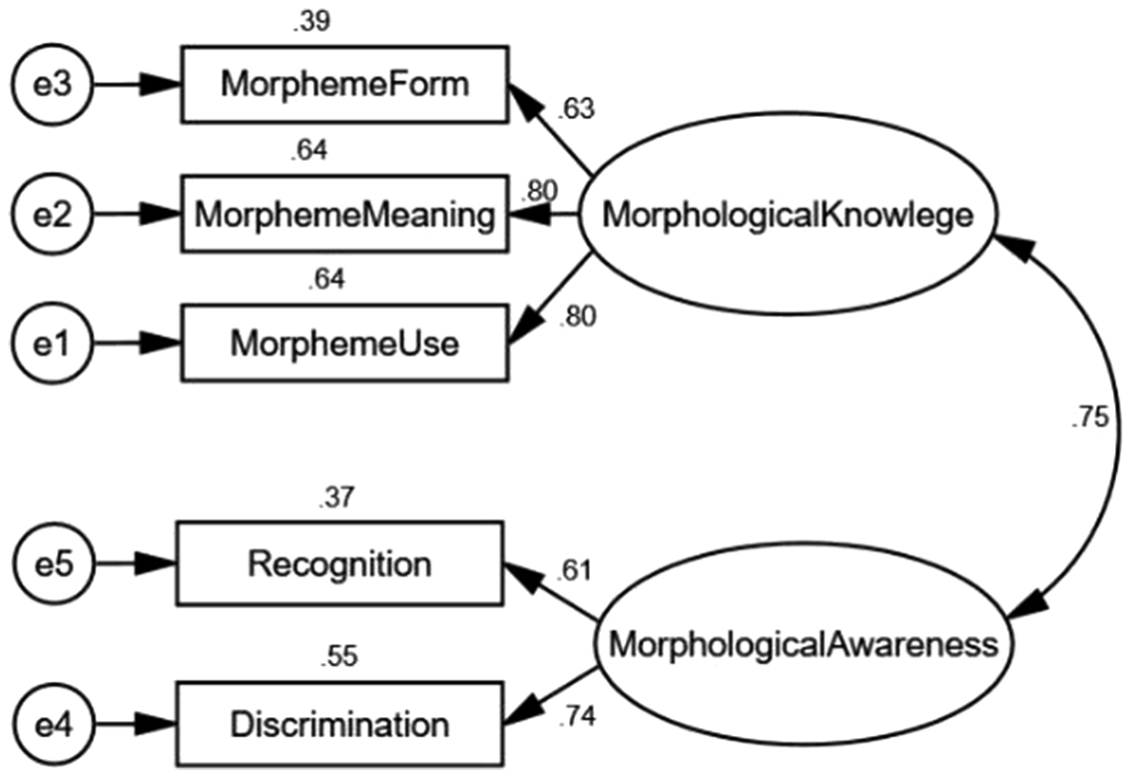
Two-factor confirmatory analysis of morphological knowledge and morphological awareness.

Pertaining to the constructs of morphological knowledge and morphological awareness, we hypothesized two measurement models. To be specific, in model 1 (Figure 1), a latent variable was created for morphological knowledge/or morphological awareness, assuming that morphological knowledge and morphological awareness were under the unitary construct whereas in model 2 as shown in Figure 2, two latent variables were created for morphological knowledge and morphological awareness, intending to test whether morphological knowledge and morphological awareness were two distinct constructs. Table 3 displays the results of model fit indices. In order to identify the best-fitting model, we compared with the criteria and relative fit indices across two models, and the results are presented in Table 3. The two-construct model [χ^2^ (4) = 8.240, CFI =0.987, TLI = 0.968, RMSEA = 0.069, ∆χ^2^ = 14.707, *p* < 0.001] demonstrated a significantly better fit to the data, which suggested that morphological knowledge and morphological awareness were two separate constructs.

**Table 3:** Model fit indexes for confirmatory factor analysis (CFA).

Model	χ^**2**^ (df)	*p*	CFI	TLI	RMSEA	*∆*χ^2^	∆df	*p*
Single-factor CFA	22.947(5)	0.000	0.945	0.890	0.126	14.707	1	0.000
Two-factor CFA	8.240(4)	0.083	0.987	0.968	0.069			

**Figure 3:**
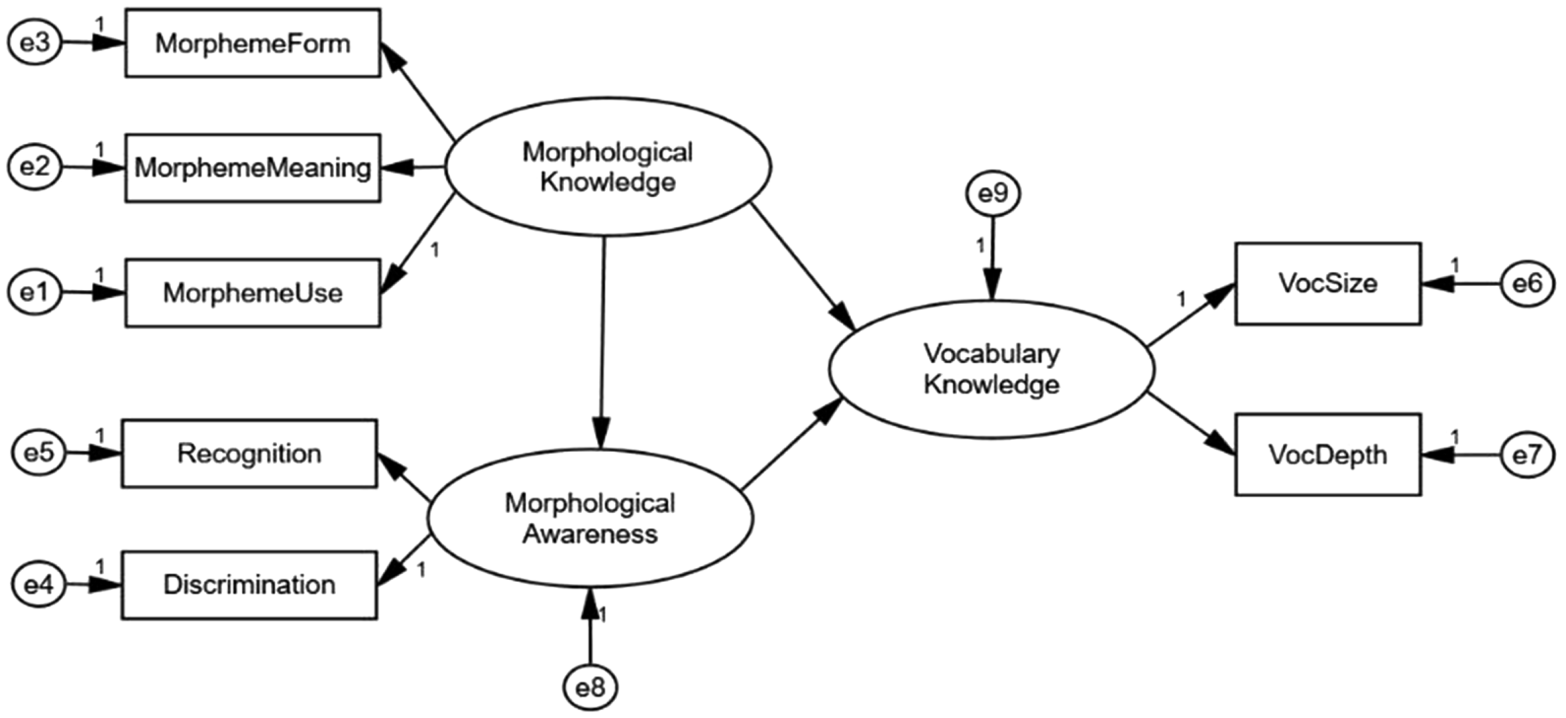
Conceptual model of the relation between morphological knowledge and vocabulary knowledge mediated through morphological awareness. Voc, vocabulary.

According to the results shown in Table 3, morphological knowledge and morphological awareness were two distinct constructs. Therefore, the model concerning the mediation through morphological awareness was tested. Based on the previous literature, morphological awareness was assumed as a unitary construct that had direct and indirect effects on vocabulary knowledge via mediators such as lexical inferencing (Wang and Zhang, 2022). The model (Figure 3) was hypothesized to test the relations between morphological knowledge and vocabulary through the mediation of morphological awareness. On the basis of the model fit indices, we found that the model hypothesizing the mediating effect through morphological awareness between morphological knowledge and vocabulary knowledge fitted the data well as evidenced by the χ^2^(11, *N* = 226) = 17.498, *p* = 0.094, CFI = 0.987; GFI = 0.978; AGFI = 0.943, RMSEA = 0.051, χ^2^/df = 1.591. In order to examine the indirect effects of morphological knowledge on vocabulary, we utilized percentile bootstrapping and bias-corrected percentile bootstrapping at a 95% confidence interval with 5,000 bootstrap samples (MacKinnon, 2008). Meanwhile, on the basis of Preacher and Hayes (2008), we also calculated the confidence interval of the lower and upper bounds to test the significance of indirect effects. As shown in Table 4, the results of the bootstrap test confirmed the existence of a positive and significant mediating effect for morphological awareness between morphological knowledge and vocabulary knowledge (indirect effect = 0.64) given that the zero is not between the bias-corrected 95% confidence interval and percentile 95% confidence interval. On the contrary, morphological knowledge made no direct effect on vocabulary knowledge because zero was between lower and upper bounds of percentile 95% CI. In the same token, the total effect was confirmed significantly as well.

**Table 4:** Results of mediating effect test.

Variables	Effect	Point estimate	Product of coefficients		Bootstrapping (5000)			
			SE	*Z*	Bias-corrected 95% CI		Percentile 95% CI	
					Lower	Upper	Lower	Upper
MK → MA → VK	Indirect effect	0.64	0.42	1.53	0.11	1.67	0.09	1.60
MK → VK	Direct effect	0.92	0.44	2.10	0.12	1.74	−0.01	1.64
MK → MA → VK & MK → VK	Total effect	1.55	0.23	6.76	1.14	2.05	1.12	2.03

## Discussion

It has been well established that morphological knowledge and morphological awareness are multidimensional (Spencer et al., 2015; Tighe and Schatschneider, 2015; Goodwin et al., 2021), and both play an important role in literacy outcomes. However, the extant literature has not made a clear demarcation between morphological knowledge and morphological awareness. The multidimensionality of morphological knowledge or awareness varied in different studies because of different components were assessed. In contrast to previous studies, the components of morphological knowledge or awareness assessed in the present study contained specific knowledge of morpheme form, morpheme meaning and morpheme use taken from prefixes and suffixes. Even though such affix knowledge has seldom studied in previous research but it necessitates morphological processing. According to Carlisle (2004), children are not likely to process a morphologically complex word without the knowledge of word parts. The findings from the present study indicated that morphological knowledge and morphological awareness were two separate constructs when specific affix knowledge was assessed as the key components. The reason why the measures of morpheme form, morpheme meaning and morpheme use were loaded on the latent variable of morphological knowledge was that morphological knowledge referred to knowledge of specific morphemes (Mitchell and Brady, 2014) while the observed variable of derivation and decomposition were loaded on morphological awareness because these two measures were intended to test learners’ sensitivity to the morphological structure of words (Carlisle, 2004). The present study used specific knowledge of morphemes together with well-accepted tasks of recognition and discrimination as components of morphological knowledge and morphological awareness, respectively, to test whether morphological knowledge and morphological awareness were same or distinct constructs. The findings showed that the standardized loadings of recognition and discrimination were 0.50 and 0.59 respectively, which fell below the ideal loading cutoff of 0.70 (Hair et al., 2013), suggesting that recognition and discrimination did not represent the construct well and they might be subsumed into another construct. In addition, the CFA results provided the indices of a good model fit and the two-factor model hypothesizing the two separate constructs of morphological knowledge and morphological awareness had the better model fit. Accordingly morphological knowledge loaded by morpheme form, morpheme meaning and morpheme use and morphological awareness loaded by recognition and discrimination were confirmed to be two separate constructs. This finding was consistent with Bialystok and Ryan’s (1985) distinction between knowledge and analysis of knowledge when they examined metalinguistic abilities. They stated that knowledge was acquired implicitly from language learning while analysis of knowledge was explicitly learned. With the increasing exposure to words and sentences, adolescent EFL students have gradually acquired tacit knowledge of morphology. The more morphemes are encountered in various contexts, the stronger the lexical representation becomes in their mental lexicon memory on the account that lexical representation is established and retrieved more quickly through tacit morphological processing (Perfetti, 2007). Meanwhile, Nagy et al. (2014) indicated that morphological instruction could have immediate and far-reaching benefits for adolescents to understand and use words through inferring the meanings of unknown morphologically complex words. Once specific morphemic knowledge is taught to adolescent EFL students, they would consciously realise that words could be broken down into morphemic units, which helps them develop the ability of strategic morphological analysis.

**Figure 4:**
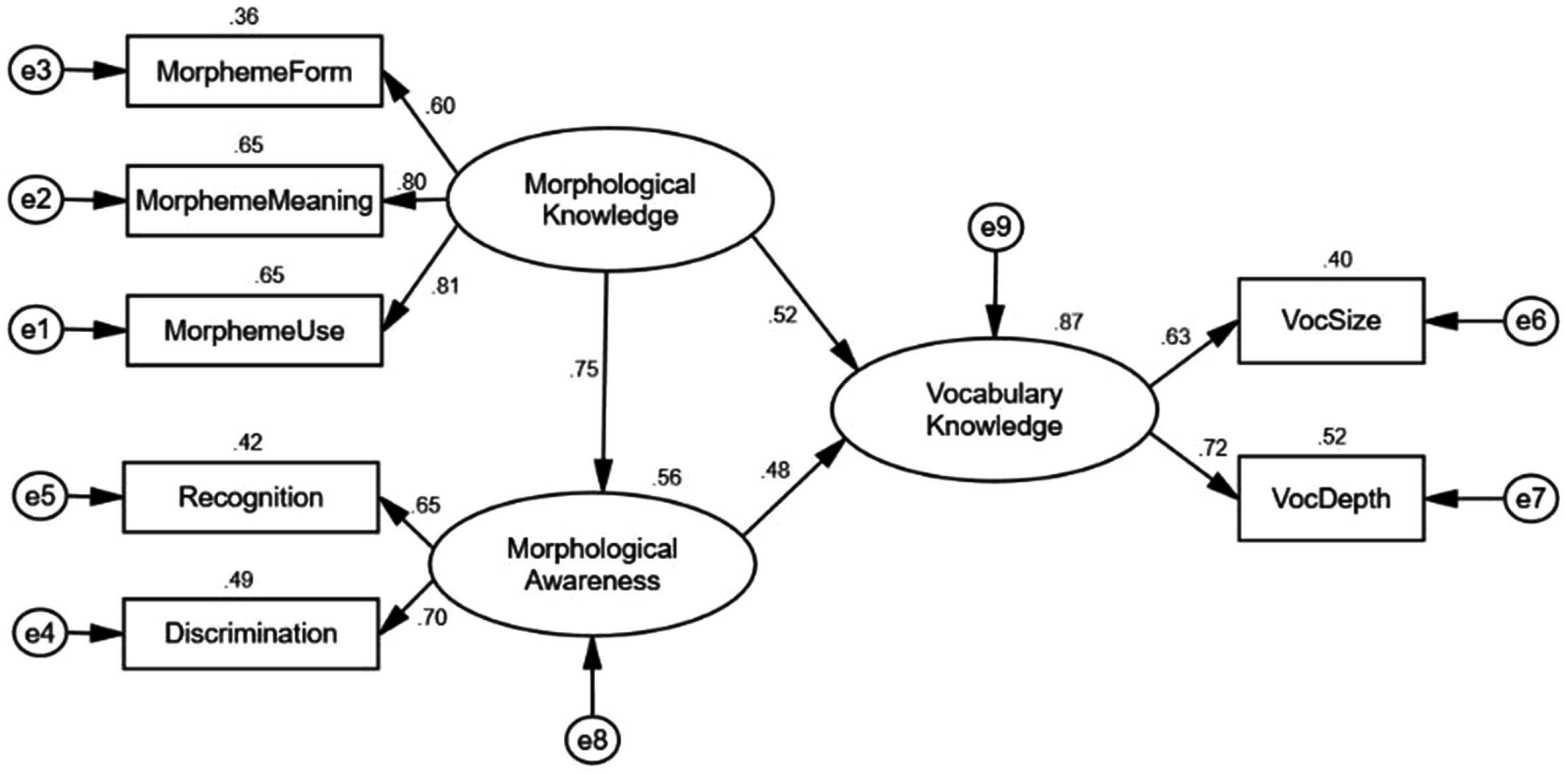
The structural model of morphological awareness in predicting vocabulary knowledge *via* morphological awareness. *p < 0.05, **p < 0.01, ***p < 0.001.

The second research question addressed the direct and indirect effects of morphological knowledge on vocabulary. Given that morphological knowledge and morphological awareness have been considered as the same construct, prior studies have evidenced the direct and indirect effects of morphological knowledge or morphological awareness on vocabulary (e.g., Anglin, 1993; Carlisle, 2000; Kuo and Anderson, 2006; Zhang and Koda, 2012). For instance, Zhang and Koda (2012) looked into the direct and indirect relationship between morphological awareness and vocabulary knowledge in a group of Chinese-speaking English language learners. It was indicated that there were significant direct and indirect relationships between morphological awareness and vocabulary knowledge, which was mediated by lexical inferencing. Wang and Zhang (2022) also investigated the effects of morphological awareness on vocabulary. It was found that morphological awareness made significant direct and indirect contributions to vocabulary size and vocabulary depth. These studies have confirmed the indirect effect of morphological awareness on vocabulary through mediating factors. However, the magnitude of effects would be influenced by component measures. The present study used specific knowledge of morphemes as indicators of morphological knowledge and recognition and discrimination as indicators of morphological awareness. As noted above, morphological knowledge and morphological awareness were confirmed as two separate constructs. The results verified that morphological knowledge had no significant direct effect on vocabulary knowledge but there was a significant indirect relationship between them which was mediated by morphological awareness (Figure 4).

It is important to consider the reason why morphological awareness could mediate the relation between morphological knowledge and vocabulary knowledge among adolescent EFL students. According to Carlisle (2004), children are not likely to process a morphologically complex word without the knowledge of word parts. It suggests that morphological awareness is built on existing morphological knowledge. Goodwin et al. (2017) indicated that through exposure to spoken and print words, young children first acquired morphological knowledge. These morphemic experiences can lead to strategic morphological processing which in turn contributes to vocabulary size and depth (Nagy et al., 2014). For adolescent EFL students in China, they have learned words through memorizing definitions and spelling since they start to learn English in third grade. As they progress to high school, they have been taught to enlarge and deepen word knowledge through analyzing the internal structure of words such as analysis of root words and affixes. Therefore, it seems that most adolescents follow a route of vocabulary learning from the formation of morphological knowledge to the utilization of morphological awareness.

In addition, it is also crucial to discuss the insignificant direct effect of morphological knowledge on vocabulary knowledge among adolescent EFL students. As indicated by Valtin (1984), adolescents move from functional use of linguistic units to deliberate analysis and manipulation of linguistic units. Before high school, students may mainly learn words through memorizing whole-word meanings. When they are high schoolers, they are taught to deliberately learn words through analyzing internal word structure to retrieve meanings of derived words. This ability to consciously use of morphological analysis is developed when adolescent EFL students become cognizant of the ways that words can be decomposed into meaningful units. Carlisle and Stone (2005) found that high school students performed faster than middle schoolers in recognizing derived words. That is, when high school students acquired more tacit morphological knowledge, they shifted more attention to strategic morphological processing while encountering unfamiliar derived words, which might explain the reason for insignificant direct effect on vocabulary knowledge.

Collectively, the present study found that morphological knowledge and morphological awareness were two separate constructs and morphological awareness significantly mediated the relation between morphological knowledge and vocabulary. Of note, the findings of the current study highlighted the importance of operationalizations in interpreting empirical results.

## Conclusion and limitations

The present study shed light on the constructs of morphological knowledge and morphological awareness as well as its relationship with vocabulary knowledge by using CFA and SEM. The results demonstrated that morphological knowledge and morphological awareness were two separate constructs. In regard to the direct and indirect effects between morphological knowledge and vocabulary, it was indicated that morphological knowledge made significant indirect contributions to vocabulary through morphological awareness. However, there was no direct effects of morphological knowledge on vocabulary knowledge. In addition, the current study has important implications to adolescents’ vocabulary learning. In regard to vocabulary instruction for EFL learners in China, first of all morphemes need to be emphasized and explicitly instructed in schools. Even though the importance of morphemic knowledge in vocabulary learning has been clearly formulated in General Senior High School Curriculum Standards (2017 edition) issued by the Ministry of Chinese Education, the specific knowledge of morphemes remains unfamiliar to many students. Secondly, teachers need to have an insight into the levels of morphemes that students should develop at different stages. It was recommended that morphemes be taught based on their frequencies. Otherwise, it will impose a heavy learning burden on students. Pertaining to the vocabulary research, the acquisition of morphemes of prefixes or suffixes needs further study. To be specific, how and when different classes of suffixes are acquired are important for instructors. In addition, the distinctions between morphological knowledge and morphological awareness depend on the measurement tasks used to assess these two concepts; therefore, future studies need to center on the acquisition of morphology across grades and the validity of different tasks so as to find out the appropriate instruments for each.

In addition to the significance of the study, there are also some limitations to take into consideration. First, the subjects were randomly sampled from two grade levels but they were considered as a whole. Future work could examine individual differences within this model. Second, the constructs of morphological knowledge and morphological awareness were determined by the type of components assessed inasmuch as different tasks tap disparate constructs. More multiple measures are suggested to be used to explore the multidimensionality of morphological knowledge. Last but not the least, the present study was conducted among students in senior high schools; as a result, the findings from the present study have limited generalizable implications for other school-age students.

## Data availability statement

The raw data supporting the conclusions of this article will be made available by the authors, without undue reservation.

## Ethics statement

The studies involving humans were approved by the ethics committee of East China Normal University (approval no. HR281–2022). The studies were conducted in accordance with the local legislation and institutional requirements. Written informed consent for participation in this study was provided by the participants’ legal guardians/next of kin.

## Author contributions

TW and HZ: conceptualization and methodology. TW: data curation, formal analysis, funding acquisition, investigation, and writing – original draft. HZ: supervision and writing – review and editing. All authors contributed to the article and approved the submitted version.

## Funding

This study was funded by the First-Class Discipline Construction of Ningxia Higher Education Institutions (Pedagogy) in 2022 for the research title: “A Study of the Internal Structure of Reading in English for Primary and Secondary School Students” (Grant No.: NXYLXK2021B10).

## Conflict of interest

The authors declare that the research was conducted in the absence of any commercial or financial relationships that could be construed as a potential conflict of interest.

## Publisher’s note

All claims expressed in this article are solely those of the authors and do not necessarily represent those of their affiliated organizations, or those of the publisher, the editors and the reviewers. Any product that may be evaluated in this article, or claim that may be made by its manufacturer, is not guaranteed or endorsed by the publisher.
